# Supplementary figures and images for: Inhibition of hepatocellular carcinoma growth via modulation of the miR-221/SOX11 axis by curcumin and berberine
Source: PeerJ. 2023 Dec 7;11:e16593. doi: 10.7717/peerj.16593 (PMC10710771; doi:10.7717/peerj.16593)

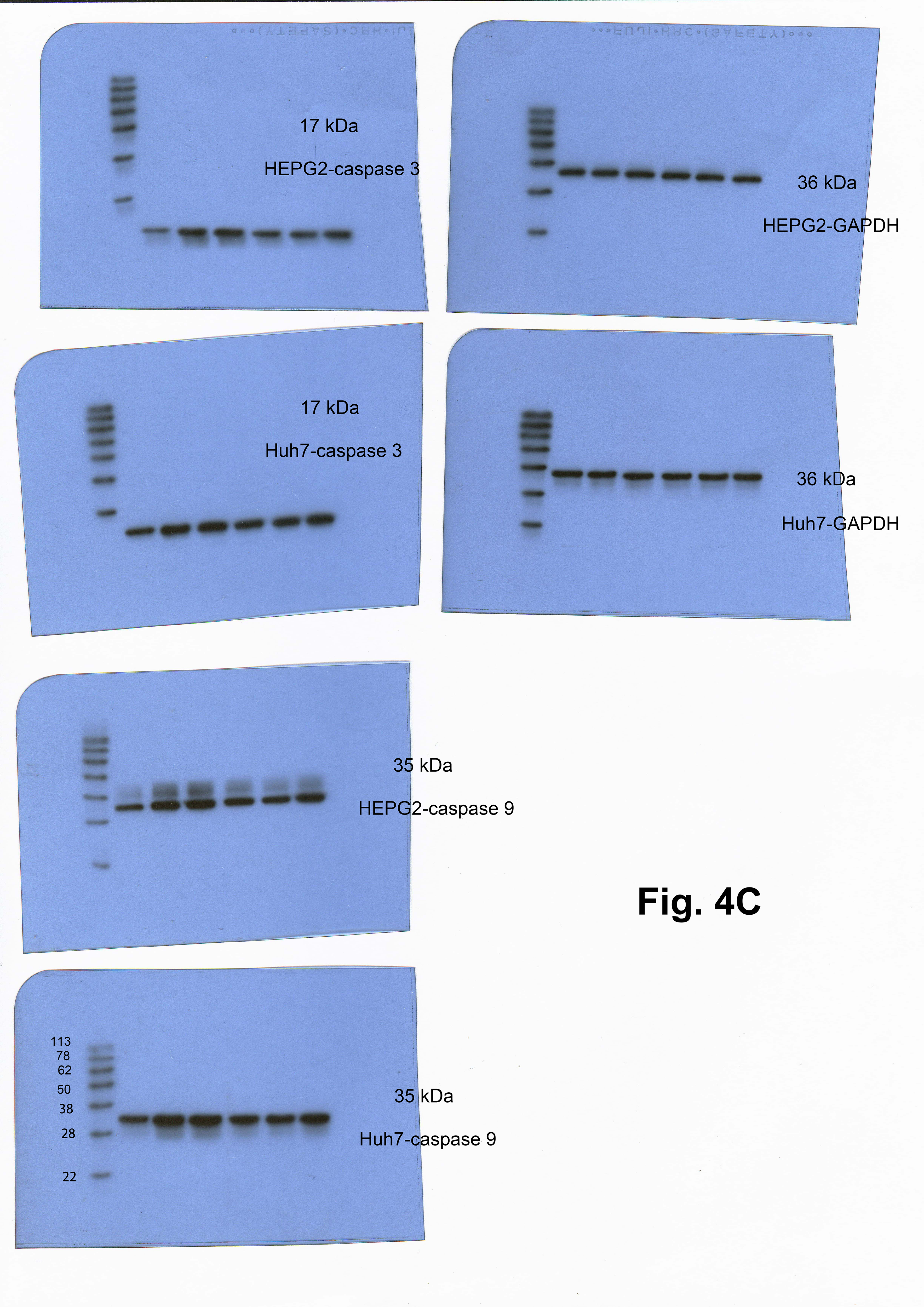

Supplement: Supplemental Information 1 [file peerj-11-16593-s001.zip › Figure 4C.tif]

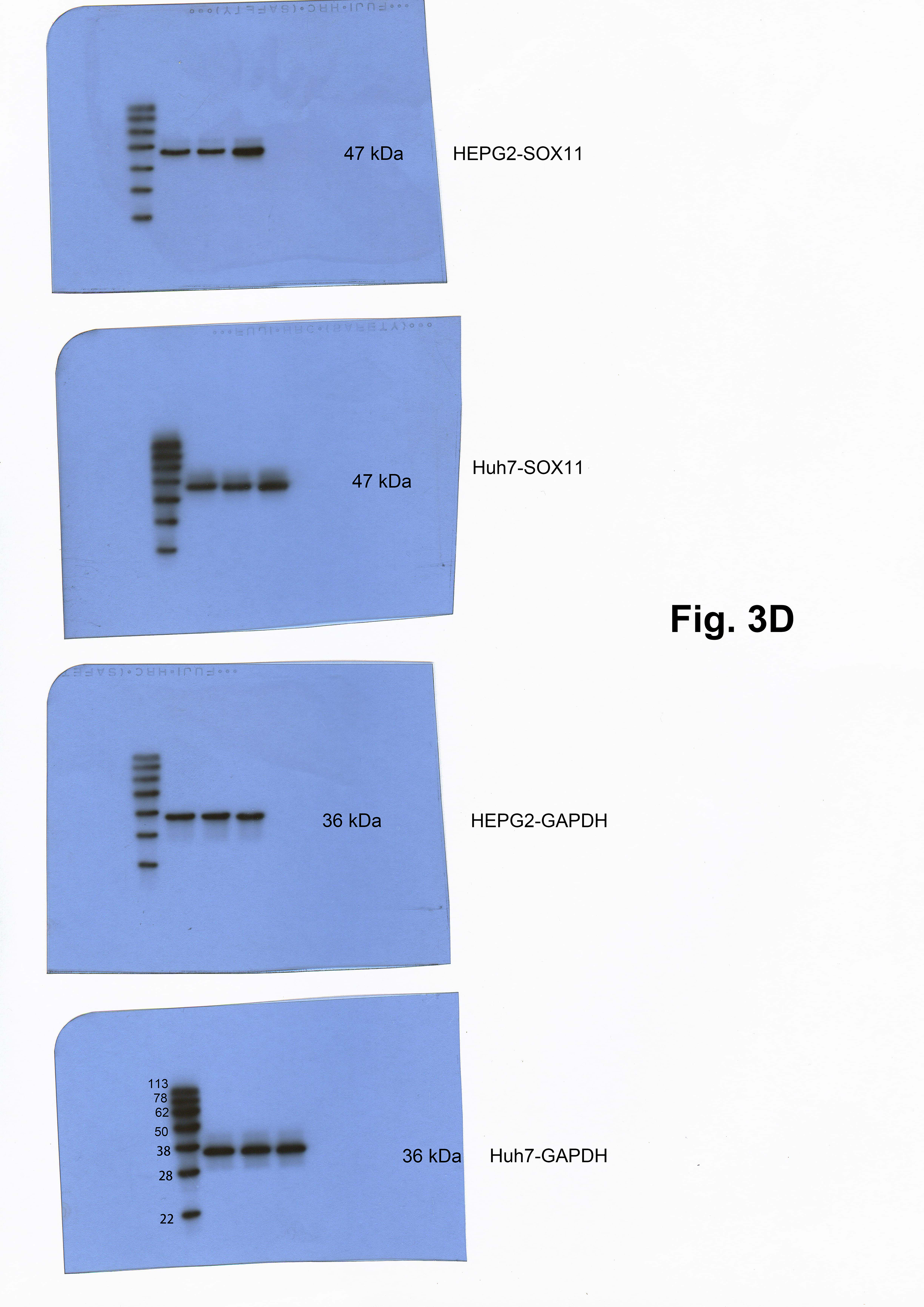

Supplement: Supplemental Information 1 [file peerj-11-16593-s001.zip › Figure 3D.tif]
