# Supplementary material for: Inhibition of hepatocellular carcinoma growth via modulation of the miR-221/SOX11 axis by curcumin and berberine
Source: PeerJ. 2023 Dec 7;11:e16593. doi: 10.7717/peerj.16593 (PMC10710771; doi:10.7717/peerj.16593)
Supplement: Supplemental Information 2 [file peerj-11-16593-s002.pdf]

MIQE Checklist

ITEM TO CHECK	IMPORTANCE	CHECKLIST
**EXPRIMNETAL DESIGN**		
Definition of experiment and control group	E	Cell culture and transfection section/line100-112
Number within each group	E	Cell culture and transfection section, line108
Assay carried out by core lab or investigator's lab?	D	Cell culture and transfection section, line108
Acknowledgement of author's contributions	D	None
Description	E	
Volume /mass of sammples processed	D	Cell culture and transfection section, line 107-108
Microdissection or macrodissection	E	None
Processing procedure	E	
If frozen-how and how quickly?	E	None
If fixed-with what how quickly?	E	None
Sample storage conditions and durarion especially for ffpe sample	E	None
**NUCLEIC ACID EXTRACTION**		
Procedure and/or details of any modifications	E	
Name of kit and details of any modifications	E	RT-qPCR assay section/line 120-132
Source of additional reagents used	D	RT-qPCR assay section/line 120-132
Details of DNase or RNase treatment	E	RT-qPCR assay section/line 120-132
Contamination assessment (DNA or RNA)	E	RT-qPCR assay section/line 120-132
Nucleic acid quantification	E	
Instrument and method	E	RT-qPCR assay section/line 120-132
Purity(A260/A280)	D	RT-qPCR assay section/line 120-132
Yield	D	RT-qPCR assay section/line 120-132
RNA integrity method/instrument	E	
RIN/RQI or Cq of 3’and 5'transcripts	E	RT-qPCR assay section/line 120-132
Electrophoresis traces	D	RT-qPCR assay section/line 120-132
Inhibition testing (Cq dilutions, spike or other)	E	RT-qPCR assay section/line 120-132
**REVERSE TRANSCRIPTION**		
Complete reaction conditions	E	RT-qPCR assay section/line 120-132
Amount of RNA and reaction volume	E	RT-qPCR assay section/line 120-132
Priming oligonucleotide and concentration	E	RT-qPCR assay section/line 120-132
Reverse transcriptase and concentrarion	E	RT-qPCR assay section/line 120-132
Temperature and time	E	RT-qPCR assay section/line 120-132
Manufacture of reagents and catalpgue numbers	D	RT-qPCR assay section/line 120-132
Cqs with and without RT	D	RT-qPCR assay section/line 120-132
Storage conditions od cDNA	D	RT-qPCR assay section/line 120-132
**qPCR TARGET INFORMATION**		
If multiplex efficiency and LOD of each assay	E	Yes
Sequence accession number	E	Gene ID: 6664
Location of amplicion	D	RT-qPCR assay section/line 120-132

Amplicon length	E	RT-qPCR assay section/line 120-132
In silico specificity screen	E	RT-qPCR assay section/line 120-132
Pseudogenes,retropsendogenes or other homologs	D	
Sequence alignmen	D	
Secondart structure analysis of amplicon	D	
Location of each primier by exon or intron	E	RT-qPCR assay section/line 120-132
What splice variants are targeted	E	RT-qPCR assay section/line 120-132
**qPCR OLIGONUCLEOTIDES**		
Primer sequences	E	RT-qPCR assay section/line 120-132
Rtprimer DB Identification number	D	None
Probe sequence	D	None
Location and identity of any modifications	E	None
Manofacture of oligonuclortides	D	None
Purification method	D	None
**qPCR PROTOCOL**		
Complete reaction conditions	E	RT-qPCR assay section/line 120-132
Reaction volume and amount of cDNA/DNA	E	RT-qPCR assay section/line 120-132
Primer,(probe),mg++,and dNTP concentrations	E	RT-qPCR assay section/line 120-132
Polymerase identity and concentration	E	RT-qPCR assay section/line 120-132
Buffer/kit identity and manufacturer	E	RT-qPCR assay section/line 120-132
Exact chemical constitution of the buffer	D	
Additives (SYBRGREENI,DMSO)	E	None
Manufacture of plates/tubes and catalog number	D	RT-qPCR assay section/line 120-132
Complete thermocycling parameters	E	RT-qPCR assay section/line 120-132
Reaction setup (manual/robotic)	D	RT-qPCR assay section/line 120-132
Manufacture of qpcr instrument	E	RT-qPCR assay section/line 120-132
**qPCR VALIDATION**		
Evidence of optimasation(from gradients)	D	RT-qPCR assay section/line 120-132
Specificity(gel,sequence,melt,or digest)	E	RT-qPCR assay section/line 120-132
For SYBR green GREEN I,cq of the NTC	E	None
Standard curves with slope and y-intercept	E	RT-qPCR assay section/line 120-132
PCR effiency calculated from slope	E	RT-qPCR assay section/line 120-132
Confidence interval for pcr efficiency or standard error	D	None
r2 of standard curve	E	RT-qPCR assay section/line 120-132
Linear dynamic range	E	None
Cq variation at lower limit	E	None
Confidence intervals throughout range	D	None
Evidence for limit of detection	E	None
If multiplex efficiency and LOD of each assay	E	None
**DATA ANALYSIS**		
qPCR analysis program (source,version)	E	RT-qPCR assay section/line 120-132
Cq method determination	E	Quantitative reverse transcription-polymerase chain
Outlier identification and disposition	E	None

Result of NTCs	E	None
Justification of number and choice of reference genes	E	None
Description of normalisation method	E	Quantitative reverse transcription-polymerase chain
Number and concordance of biological replicates	D	None
Number and stage (RT or qpcr)of technical replicates	E	Quantitative reverse transcription-polymerase chain
Repeatability (intra assay variation,)	E	None
Repeatability (intra assay variation,%CV)	D	None
Power analysis	D	None
Statistical methods for result significance	E	Statistical analysis section/line 165-168.
Software(source,version)	E	Statistical analysis section/line 165-168.
Cq or raw data submission using RDML	D	None
